# Flip-flop Converter of Dual-bistability Using Cavity and Parametric Amplified Four-Wave Mixing

**DOI:** 10.1038/s41598-018-20962-5

**Published:** 2018-02-06

**Authors:** Kangkang Li, Renan Bu, Xiuxiu Wang, Haixia Chen, Dan Zhang, Xinghua Li, Yanpeng Zhang

**Affiliations:** Key Laboratory for Physical Electronics and Devices of the Ministry of Education & Shaanxi Key Lab of Information Photonic Technique, Xi’an Jiaotong University, Xi’an, 710049 China

## Abstract

We study the realization of dual-bistability flip-flop converter in cavity and parametrically amplified four-wave mixing (FWM) process at a four-level cavity atomic system. Using the effect of nonreciprocity optical dual-bistability, we can obtain different output multi-mode states of probe transmission signal and FWM signal. We find the channel equalization ratio and optical contrast between multi-mode states is related to the degree of dual-bistability. Besides, the degree of dual-bistability can be controlled by the input parameters (frequency detuning and powers of the dressing beams). More, using electro-optical modulator and acoustic optical modulator to modulate the powers and frequency detuning, respectively, we can realize the fast conversion between different output states. And the switch speed of this flip-flop converter is about 16 ns. These outcomes may provide foundation for the development of all-optical devices and quantum information processing.

## Introduction

In the last few years, a great deal of optical phenomena based on atomic coherence and quantum interference have attracted intensive attention of many researchers in the multilevel atomic systems. Four-wave mixing (FWM) processes due to atomic coherence have been experimentally reported in all alkali atoms^1–6^ and various experimental schemes^7–9^. With widely studied in applications, FWM plays potential roles in the sub shot noise measurements^1,10,11^, quantum imaging^12,13^, quantum communication^14^, slow light^15^, storage of light^16^ and relative intensity squeezing^5^. Besides, with the self-stabilizing function, FWM can cause the energy transfer of different waves^17–19^. A spontaneous parametric four wave mixing (SP-FWM) process that generates two weak fields can amplify the seeded signal, the phenomenon is called optical parametrically amplification (OPA)^20,21^. More, optical bistability (OB) behavior based on atomic coherence and quantum interference are practiced in recent decades^22,23^. On the other hand, some schemes for realizing optical stability through multi-wave mixing process in an optical cavity have been studied experimentally and theoretically^15^, where cavity can provide a feedback as an essential factor for the generation of bistability^24^. OB has also been demonstrated without a cavity using degenerate FWM in atomic vapor with two counter-propagating laser beams^25,26^. The OB in multilevel atoms inside optical cavities^27,28^ has been the subject of many recent studies because of its broad application prospects in all-optical logic and memories performance^29,30^. Bistability and instability were also observed in cold clouds of cesium atoms inside an optical cavity, where degenerate Zeeman sublevels participate in the dynamic processes^31^.

In this paper, we investigate the nonreciprocity optical dual-bistability (ODB) phenomena of probe transmission signal (PTS) and four-wave mixing (FWM) signal in a composite atom-cavity system both theoretically and experimentally. Based on the relationship of vacuum Ribi splitting and OB^24^, the ODB is obtained by scanning the frequency detuning of beams, which is more sensitive than scanning the powers. The nonreciprocity of frequency (frequency offset, in *x* direction) and intensity (shape change, in *y* direction) between the two signals (from the rising and falling edges in one frequency scanning round trip) are attributed to “∞”-shape non-overlapping region^32^, which can reflect the degree of dual-bistability directly. Besides, the frequency offset is caused by the cavity feedback dressing effect and has been amplified by the effect of vacuum induced enhancement in cavity^24^. Moreover, the nonlinear refractive index and the enhancement and suppression of FWM cavity mode have been studied. By changing the frequency detuning and the powers of dressing beam, the feedback intensity will be enhanced or suppressed, which results in many kinds of multi-mode output states of signals. The ODB in FWM process provide a solution for generating the multi-mode output states at a time, which means the signals can convey more information. With the controlling of switch between different output states, the action of this phenomena can be realized as a dual-bistability flip-flop converter. These results may have novel and promising development for generation and potentially applicable in all-optical devices and quantum information processing.

## Experimental setup and Basic theory

**Figure 1:**
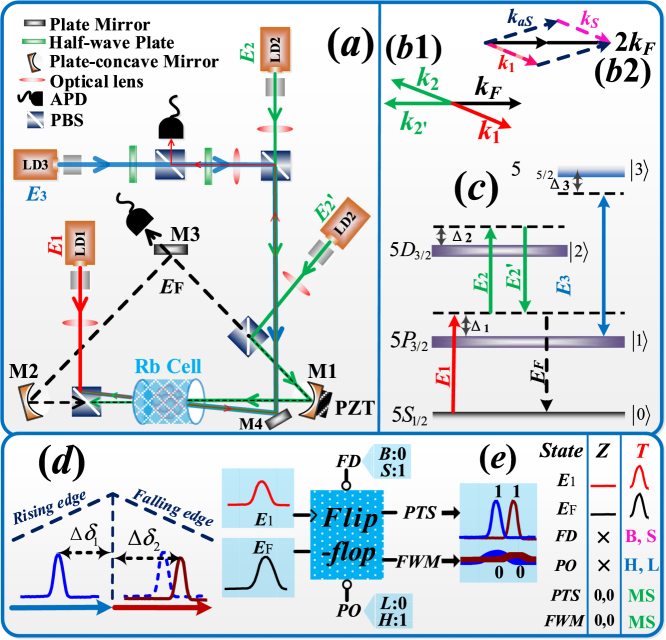
(**a**) Experimental setup. PBS: polarization beam splitter; APD: avalanche photodiode detector; PZT: piezoelectric transducer, which can control the length of cavity; LD: laser device; M1: plate-concave mirror; M2: plate-concave mirror; M3: plate mirror; M4: high reflectivity mirror. (**b1**) Phase-matching geometrical diagram of the cavity-FWM processes; (**b2**) Phase-matching geometrical diagram of the PA-FWM processes. (**c**) Energy-level diagram for the laser coupling configuration in ^85^Rb vapor. (**d**) The “∞”-shape non-overlapping region between the two signals (from the frequency-rising and frequency-falling edges in one frequency scanning round trip). (**e**) Schematic diagram of dual-bistability flip-flop. FD: frequency detuning; B: big; S: small; PO: power; L: low; H: high; Z: zero; T: trigger; X: arbitrary value; MS: multi-mode states.

A composite atom-cavity system contains a four-level ^85^Rb atomic vapor cell in the optical ring cavity. The details of the experimental setup are described in the **Methods** section. The four relevant energy levels are 5*S*_1/2_ (|0>), 5*P*_3/2_ (|1>), 5*D*_3/2_, (|2>), and 5*D*_5/2_, (|3>) as show in Fig. 1(c). In this system, a weak probe beam ***E***_1_ (frequency *ω*_1_, wave vector ***k***_1_, Rabi frequency *G*_1_, vertically polarized) couples the transition |0> to |1>, while a strong pumping beam ***E***_2_ (*ω*_2_, ***k***_2_, *G*_2_, horizontally polarized) and another pumping beam ***E***_2’_ (*ω*_2’_, ***k***_2’_, *G*_2’_, vertically polarized) couples upper transition |1> to |2>. Here the detuning Δ_*i*_ = Ω_*i*_ − *ω*_*i*_ is defined as the difference between the resonant transition frequency Ω_*i*_ and the laser frequency *ω*_*i*_ of ***E***_*i*_. Besides, ***E***_1_ counter-propagates with ***E***_2_, and ***E***_2’_ propagates along the optical axis of the cavity having an angle of 2° with ***E***_2_ as show in Fig. 1(a). The external-dressing beam ***E***_3_ (*ω*_3_, ***k***_3_, *G*_3_) propagates along ***E***_2_ direction and drives the other upper transition |1> to |3>. When all incident beams are focused at the center of the rubidium cell by optical lenses, a phase conjugate FWM signal ***E***_*F*_ (*ω*_*F*_, ***k***_*F*_, *G*_*F*_, horizontally polarized) is satisfying the phase-matching condition (***k***_*F*_ = ***k***_1_ + ***k***_2_ − ***k***_2’_ as show in Fig. 1(b1)) and propagates in the opposite direction of ***E***_2’_, which means the signal of ***E***_*F*_ is mode-matched to the cavity and can form cavity mode. The cavity transmission spectrum of ***E***_*F*_ leaked from M3 is detected by an avalanche photodiode detector (APD). According to the energy system and Liouville pathways, the generated ***E***_*F*_ can be obtained by solving the density-matrix equations, the third-order density matrix element as follow:

1 $${\rho }_{10{\rm{F}}}^{(3)}=(i{G}_{1})(i{G^{\prime} }_{2})(i{G}_{2})/({{d}_{1}}^{{\rm{2}}}{d}_{2}).$$

where *d*_1_ = Γ_10_ + *i*Δ_1_, *d*_2_ = Γ_20_ + *i*(Δ_1_ + Δ_2_), *G*_*i*_ = *μ*_*ij*_***E***_*i*_/ħ (*i*, *j* = 1, 2, 2’) is the Rabi frequency between levels |*i*>→|*j*>, and *μ*_*ij*_ is the dipole moment; Γ_*ij*_ = (Γ_*i*_ + Γ_*j*_)/2 is the decoherence rate between |i> and |j>. Then, the generated ***E***_*F*_ field can oscillate and circulate inside the three-mirrors ring cavity while ***E***_1_, ***E***_2_ and ***E***_2’_ cannot, which is caused by the direction of angle and effect of PBS. Besides, if the atomic transition |0>→|1> is resonant with cavity and the atom cavity coupling is strong enough, the atom-cavity coupling needs to be considered in the system. By solving the master equation of the cavity field evolution and the density matrix operators (the details of the solving process are described in the **Methods** section), we can get the cavity mode of ***E***_F_ by solving master equation as:

2 $${a}_{FWM}=-g\sqrt{N}{G}_{F}/[{d}_{4}({d}_{1}+{g}^{2}N/{d}_{4}+{|{G^{\prime} }_{2}|}^{2}/{d}_{2}+{|{G}_{3}|}^{2}/{d}_{3}].$$

where *d*_3_ = Γ_30_ + *i*(Δ_1_ + Δ_3_); *d*_4_ = *γ* + *i*(Δ_1_ −  Δ_*ac*_); *g* is the single-atom-cavity coupling strength and *N* is the atom number; *gN*^1/2^ represents the atom–cavity coupling which is caused by the combined action between resonant fluorescence of ***E***_1_ and vacuum induced enhancement of FWM; the term *g*^2^*N*/*d*_4_ represents the cavity dressing effect; the terms |*G*^′^_2_|^2^/*d*_2_ and |*G*^′^_3_|^2^/*d*_3_ are the internal-dressing effect of ***E***_2’_ and ***E***_3_, respectively.

Specifically, by considering FWM process in the atom-cavity system with strong self-Kerr nonlinear effect, there exists an un-neglected cavity feedback effect (also a self-dressing effect)^24^. Clearly, the generated ***E***_F_ field have a self-dressing effect of |*G*_F_|^2^, which is derived from the relatively strong feedback effect. This self-dressing effect have similar influence with ***E***_1_, ***E***_2’_, ***E***_3_ and *g*^2^*N*, so Eq. (2) can be rewritten as:

3 $${a}_{FWM}=-g\sqrt{N}{G}_{F}/[{d}_{4}({d}_{1}+{g}^{2}N/{d}_{4}+{|{G^{\prime} }_{2}|}^{2}/{d}_{2}+{|{G}_{3}|}^{2}/{d}_{3}+{|{G}_{F}|}^{2}/{{\rm{\Gamma }}}_{00})].$$

Besides, the probe beam ***E***_1_ has a sufficiently low power, as well as is far detuned from |0>→|1>. Moreover, with enhanced cavity mode of ***E***_F_, a SP-FWM process will occur in the system, which can generate two weak fields (Stokes field ***E****s* and anti-Stokes field ***E****as* which satisfying the phase-matching condition 2***k***_*F*_ = ***k****s* + ***k****as*). Therefore, the ***E***_1_ is naturally injected into the input Stokes or anti-Stokes port of the SP-FWM process as shown in Fig. 1(b2), and the injection will serve as an OPA process (with phase-matching conditions ***k***_*aS*_ = 2***k***_*F*_ − ***k***_1_ and ***k***_*S*_ = 2***k***_*F*_ − ***k***_1_) assisted by the cascaded nonlinear process. The signal of ***E***_1_ beam that amplify by OPA-FWM process is named as PTS, and it is detected by the other APD. The photon numbers of the output Stokes and anti-Stokes fields in the amplification process with injection are described in the **Methods** section.

Further, by turning on probe and coupling fields, the first-order density matrix element with consideration of the dressing effects from ***E***_1_, ***E***_3_ and ***E***_*F*_ is given via Liouville pathways:

4 $${\rho }_{10}^{(1)}=i{G}_{1}/({d}_{1}+{{G}_{1}}^{2}/{{\rm{\Gamma }}}_{00}+{{G}_{3}}^{2}/{d}_{3}+|{G}_{F}^{A}{|}^{2}/{{\rm{\Gamma }}}_{00}).$$

What’s more, similarly to the proposed concept of vacuum induced transparency, the vacuum induced nonreciprocity responses for PTS and FWM are caused by |*G*_*F*_|^2^. As this cavity feedback dressing term |*G*_*F*_|^2^ is not equal between the signal curves in the frequency-increasing and frequency-decreasing processes (corresponding to the rising and falling edges in one frequency scanning round trip, respectively), the generated nonreciprocity ODB of folded signals could exist “∞”-shape non-overlapping region for scanning the frequency detuning of probe or cavity, which includes the frequency offset in *x* direction and the shape change in *y* direction. At first, there exists frequency offset (Δ*υ*) between the two peaks or dips in the same baseline. Whereas the nonreciprocity can be interpreted by the change of nonlinear refractive index Δ*n*’, which is given as:

5 $${\rm{\Delta }}n\text{'}=N({n}_{2up}{I}_{up}-{n}_{2down}{I}_{down})={\rm{\Delta }}\sigma c/{\omega }_{p}l.$$

where term Δ*σ* = Δ*υn1l*/*c* is the phase delay and Δ*υ* is the frequency difference that can reflect the ODB phenomenon directly; I_*up*_ (I_*down*_) is the feedback intensities of the signals for the PTS and FWM on the two side ramps generated at the same frequency scan. n_2*up*_ (n_2*down*_) is the nonlinear refractive index coefficient that can be generally expressed as *n*_2*up*_ ≈ *n*_2*down*_ ≈ *n*_2_=Re[*χ*^(3)^/(*ε*_0_*cn*_0_)], which is the mainly dominated by the Kerr coefficient of ***E***_2_. The nonlinear susceptibility is:

6 $${\rm{\Delta }}n\text{'}=N{n}_{2}({I}_{up}-{I}_{down})={\rm{\Delta }}\sigma c/{\omega }_{p}l.$$

Besides, the shape change of the signals can also advocate the ODB effect in the composite cavity-atom system which can be understood through requirement for the dressing suppression and enhancement. When considering the different feedback dressing on the rising and falling edges, the signal will meet different enhancement (or suppression) conditions. For instance, when we scan Δ_3_, the primary A-T splitting is caused by ***E***_3_ whose corresponding eigenvalues are *λ*_±_ = [∆_3_ ± (∆_3_^2^ + 4|*G*_3_|^2^)^1/2^]/2, so the suppression and enhancement conditions of *G*_3_ are ∆_1_ + ∆_3_ = 0 and ∆_1_ + λ_±_ = 0, respectively. The secondary A-T splitting is caused by the feedback dressing term *G*_*F*_ whose corresponding eigenvalues are *λ*_+±_ = [∆_3_'^2^ ± (∆_3_'^2^ + 4|*G*_*F*_|^2^)^1/2^]/2 (∆_3_′ = −∆_1_−*λ*_+_), and the suppression and enhancement conditions are ∆_1_ + ∆′_3_ = 0 and ∆_1_ + λ_±_ + *λ*_+±_ = 0, respectively. Further, when we scan Δ_*ac*_, the split energy levels are *λ*_+±_ ± *gN*^1/2^ and *λ*_−_, where *λ*_++±_ = [∆_*ac*_′ ± (∆_*ac*_′ + 4*g*^2^*N*)^1/2^]/2 (∆_*ac*_′ = −∆_*ac*_ − *λ*_+±_), the respective suppression and enhancement conditions are ∆_1_ + ∆_*ac*_′ = 0 and ∆_1_ + λ_±_ + *λ*_+±_ + *λ*_++±_ = 0.

## Results and Discussion

In our experiment, we investigate the multi-mode output states that is modulated by the different input parameters. Firstly, we obtain the nonreciprocity of the probe transmission signal (PTS) and the cavity mode four-wave mixing (FWM) signal by scanning the frequency detuning of external-dressing field ***E***_3_ (Δ_3_) or cavity length (namely, ∆*ac*). The cavity length is controlled by the PZT (connecting with plate-concave mirror M1) at on left direction is “frequency-rising edge” and at the other direction is “frequency-falling edge” in Fig. 1(a1). That signals come from electromagnetically induced transparency (EIT) window ∆_1_ + ∆_2_ = 0. The curves of PTS perform the combination of electromagnetically induced absorption (EIA) dip and gain peak, which can express as ${I}_{p}\propto ({I}_{0}-\text{Im}{\rho }_{10}^{(1)}+{|{\rho }_{01}^{(3)}|}^{2})$ (where *I*_0_ is the intensity of the probe field without Doppler absorption; *ρ*^(3)^_01_ and *ρ*^(1)^_10_ (in Eq. (6)) show as gain peak and EIA dip, respectively). Meanwhile, the intensity of cavity mode FWM signal is related to *a*_*FWM*_ in Eq. (3). In Fig. 1(d), with scanning Δ_3_, the left and right curves of the bell shaped represent the frequency-rising edge and frequency-falling edge, respectively, and the vertical curve represents the turning point of the round trip. Correspondingly, there are the signals of F cavity mode FWM, where the left (right) peaks belong to the signals of rising (falling) edge in one frequency scanning round trip. Besides, the frequency detuning between left peak and turning point *T* is ∆*δ*_1_, and the frequency detuning between right peak and *T* is ∆*δ*_2_. Therefore, when fold the signals on the two edges (rising and falling) from the maxima of the ramp curves point *T* do not overlap, the frequency offset can express as *δ* = ∆*δ*_2_ − ∆*δ*_1_. In our results, we also use this way (*δ* = ∆*δ*_2_ − ∆*δ*_1_) to analyze the frequency offset. Besides, it is obvious that the shape of right peak is different from left peak. Therefore, the degree of nonreciprocity can be demonstrated by non-overlapping region includes frequency offset and shape change which can be approximately viewed with infinite sidebands, so we named this kind of nonreciprocity as “∞”-shape ODB.

**Figure 2:**
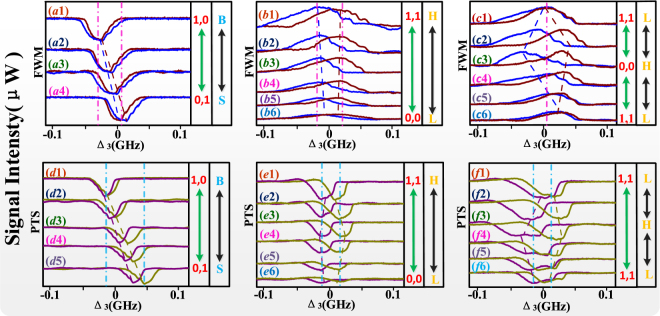
All the experiment curves are “T” (trigger) state; B: big; S: small; L: low; H: high. 0,0: output state of signals; 1,1: output state of signals. 0,1: output state of signals; 1,0: output state of signals. (**a**–**c**) and (**d**–**f**) measured cavity mode (FWM) and PTS, respectively. (**a**,**d**) are obtained against versus Δ_3_ at discrete Δ1 from “B” state to “S” state. (**b**,**e**) are obtained against versus Δ_3_ at different points of ***E***_3_ power from “H” state to “L” state. (**c**,**f**) are obtained against versus Δ_3_ at different points of ***E***_1_ power from “L” state to “H” state then to “L” state.

**Figure 3:**
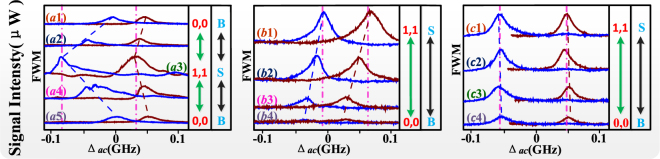
All the experiment curves are “T” (trigger) state by measuring cavity mode (FWM) with beam ***E***_3_ blocked; B: big; S: small; 0,0: output state of signals; 1,1: output state of signals. In a period, (**a**) versus Δ_*ac*_ at discrete Δ_1_ from “B” state to “S” state then to “B” state; (**b**) versus Δ_*ac*_ at discrete Δ_1_ in a half period (from “S” state to “B” state); (**c**) versus Δ_*ac*_ at discrete Δ_2_ from “S” state to “B” state.

**Figure 4:**
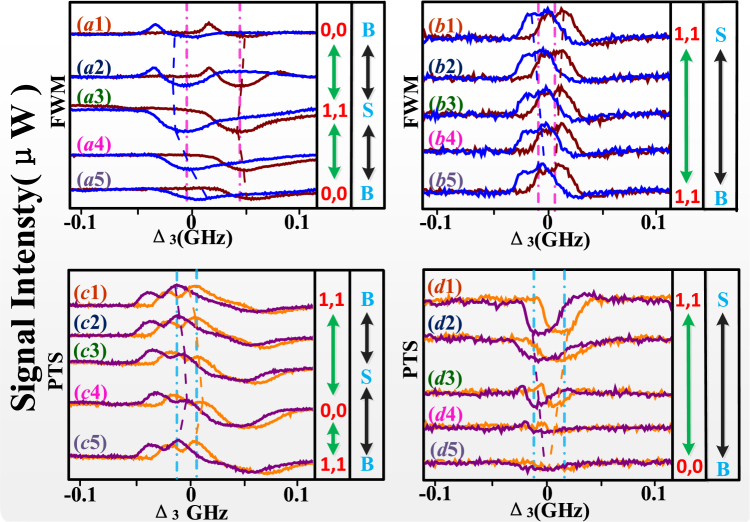
All the experiment curves are “T” (trigger) state by measuring cavity mode (FWM) and PTS; B: big; S: small; 0,0: output state of signals; 1,1: output state of signals. (**a**) and (**c**) are obtained against versus Δ_3_ at discrete Δ1 from B-state to S-state then to B-state. (**b**,**d**) are obtained against versus Δ_3_ at discrete Δac from S-state to B-state.

Secondary, the action of ODB phenomena can be realized as a dual-bistability flip-flop converter and schematic diagram as show in Fig. 1(e), where ***E***_1_ is trigger signal, ***E***_*F*_ is input signal, FD (frequency detuning) and PO (power) are input parameters, PTS and FWM are output multi-mode states. There also have two states (“zero” state and “trigger” state) of this flip-flop and it mainly works at “trigger” state. FD is controlled by an electro-optical modulator and the speed is 10 *ns*, while PO is controlled by an acoustic optical modulator and the speed is 12 *ns*. The switching speed is controlled by the atomic coherence time that is mutable by the phonon effect from microseconds to nanoseconds. The total switching speed (16 *ns*) of this flip-flop converter is taken to be the quadrature sum of several independent contributions. In following experimental results (Figs 2–4), with changed input parameters, we realize the fast conversion between different output multi-mode states by analyzing “∞-shape non-overlapping region and optical contrast of folded signals.

In Fig. 2, we discuss the output multi-mode states of the PTS and cavity mode FWM signal (***E***_*F*_) versus the frequency detuning of ***E***_3_ (Δ_3_). By changing frequency detuning (FD) and power (PO) of dressing fields, we realize the switch action between different output multi-mode states. In Fig. 2(a), the signal of ***E***_*F*_ is measured when versus Δ_3_ at discrete Δ_1_, where the dip of signal curve is lower than the baseline that represents suppression effect of ***E***_3_. The curves of FWM signal is related to *a*_*FWM*_ in Eq. (3). The dip gets the maximum value in Fig. 2(a4) where satisfies window ∆_1_ + ∆_2_ = 0 and Δ_1_ + Δ_3_ = 0 together, which means the value of ∆_2_ is equal to Δ_3_. Since the two peaks in Fig. 2(a) have the same baseline, the cavity feedback dressing (also a self-dressing) term |*G*_*F*_|^2^/Γ_00_ on the rising and falling edges are not equal in Eq. (3). Hence, the feedback intensity *I*_*up*_ is not equal to *I*_*down*_ while *n*_2*up*_ ≈ *n*_2*down*_, which can lead to the occurrence of frequency offset (Δ*υ*) of ODB. From top to bottom, with the increased detuning Δ_1_, the Δυ appeared larger, which is attributed to increased *n*_2_ in Eq. (6), and the Δ*υ* attains maximum value of 6.7 MHz in Fig. 2(a4) where ∆1 = 0 MHz. In this output state, with H-state of PO, FD is changed from B to S, and the output state of FWM switches from “1,0” to “0,1”. Succinctly, the optical contrast for switching application can be defined as *C* = (*I*_1_ − *I*_0_)/(*I*_1_ + *I*_0_), where *I*_1_ is the intensity of “1”-state and *I*_0_ is the intensity of “0”-state. The optical contrast calculated for this case is 100%. Since, it is obvious from the same baseline that the shape of peaks is different in Fig. 2(a), which is caused by the different suppression conditions. Here we consider second-order splitting caused by ***E***_3_ and *G*_*F*_ that we have been illustrated theoretically. Because of different *G*_*F*_ on the two edges, the suppression conditions ∆_1_ + ∆′_3_ = 0 of two peaks are not same while there are different corresponding eigenvalues *λ*. And the change of shape difference is minute from top to bottom. While Fig. 2(b,c) are the cavity modes of FWM signals by scanning Δ_3_ at different external-dressing field power (*P*_3_) and probe power (*P*_1_), respectively. Comparing with Fig. 2(a), the signals perform gain peak caused by strong enhancement of cavity-dressing effect of *gN*^1/2^ in Fig. 2(b,c). There frequency offset Δ*υ* is also attributed to cavity feedback dressing term |*G*_*F*_|^2^/Γ_00_. From bottom to top, with increased *P*_3_, both Δ*υ* and shape difference are increasing gradually in Fig. 2(b), which means the power of external-dressing field can alter the state of FWM. Where PO is changed from L to H and the state of FWM switches from “0,0” to “1,1”, while the state of FD is B, and the optical contrast calculated for this case is 78%. But in Fig. 2(c), from bottom to top, the change of Δ*υ* increases from minimum to maximum in Fig. 2(c4)–(c2), then it becomes minimum again in Fig. 2(c1), it also attributes to changed *P*_1_, which is increased first and then is decreased. In this state, with S-state of FD, PO is changed from L to H and then to L, and the state of FWM switches from “1,1” to “0,0” and then to “1,1” again. The optical contrast calculated for this case is 69%.

In Fig. 2(d–f), there are signals of PTS versus Δ_3_ corresponding to Fig. 2(a–c), respectively. The signals are only dip visually, since the suppression is much strong. Same with cavity mode FWM signal, the nonreciprocity ODB of PTS also is induced by cavity feedback dressing term (also parametrically amplified effect) |*G*_*F*_|^2^/Γ_00_ in Eqs (3) and (4). In Fig. 2(d), with the Δ_1_ increased from top to bottom, the *n*_2_ is increased, the signals in Fig. 2(d5) get the maximum value where Δ_3_ around 0 MHz. And the state of PTS also switches from “1,0” to “0,1”. In Fig. 2(b,c), all signals satisfy the enhancement condition ∆_1_ + λ_±_ + *λ*_+±_ = 0. With the decreasing of *P*_3_ and *P*_1_ in Fig. 2(e1–e6) and Fig. 2(f1–f6), the signal intensity of PTS is increased by the dressing term *G*_3_^2^/*d*_3_ in Eq. (2) and *G*_1_^2^/Γ_00_ in Eq. (6), respectively. Besides, the change process of “∞”-shape non-overlapping region in Fig. 2(e,f) are same with Fig. 2(c). In Fig. 2(e3), the Δ*υ* gets maximum value at 31.8 MHz with the largest *n*_2_. For same reason, the Δ*υ* gets maximum value at 20.1 MHz in Fig. 2(f3). After that, with the decreasing of *P*_3_ or *P*_1_, the effect of cavity feedback dressing becomes weaken. In these two cases, the states of PTS are switched from “0,0” to “1,1” and “1,1” to “1,1”, respectively. And the optical contrasts are more than 90%.

In Fig. 3, with beam ***E***_3_ blocked, we discuss the output multi-mode states of the cavity mode FWM signal (***E***_*F*_) versus the frequency detuning of cavity (Δ_*ac*_). By changing frequency detuning of dressing fields, we realize the switch action between different output multi-mode states. Same with Fig. 2, we recognize the state by analyzing the “∞”-shape non-overlapping region of folded signals.

In Fig. 3(a,b), with beam ***E***_3_ off, we measured ***E***_*F*_ versus Δ_*ac*_ at discrete Δ1, where the ***E***_*F*_ signal comes from EIT window Δ_1_ + Δ_2_ = 0. In Fig. 3(a), we change the detuning Δ_1_ in a period but in Fig. 3(b) is just half period. When the detuning Δ_1_ is adjusted the position of resonant peaks shifts in the direction of $d{{\rm{\Delta }}}_{ac}/d{{\rm{\Delta }}}_{1}=2/[1-{G}_{4}/{({G}_{4}^{2}+4{|g\sqrt{N}|}^{2})}^{1/2}]$, and the peak gets the maximum value in Fig. 3(a3). Here we changed the detuning Δ2 = 0 satisfying the conditions Δ_1_ − Δ_*ac*_ = 0 and Δ_1_ − Δ_2_ = 0, and the window Δ_1_ − Δ_*ac*_ = 0 comes from dressing effect of *gN*^1/2^ by the term *g*^2^*N*/*d*_4_ in Eq. (3). This equation manifests a positive correlation between Δ_*ac*_ and Δ_1_ with a large moving speed satisfying |*d*Δ_*ac*_/*d*Δ_1_|>2. Same with Fig. 2, the different feedback dressing parts between *I*_*up*_ and *I*_*down*_ result in the nonreciprocity of signals. In Fig. 3(a1–a3), the detuning Δ_1_ increased first then decreased in Fig. 3(a3–a5). In this case, *n*_2_ is changed from increased to decrease accordingly. The corresponding frequency offset Δυ are 49.1 MHz, 113.9 MHz and 47.0 MHz in Fig. 3(a1,a3,a5), respectively. In this state, with L-state of PO, FD of Δ_1_ is changed from S state to B state and then to S state again, and the state of FWM switches from “0,0” to “1,1” and then to “0,0” again. The optical contrast calculated for this case is 79%. While in Fig. 3(b), from top to bottom, the decreased detuning Δ_1_ result the *n*_2_ to be decreased and corresponding Δυ is changed from 66.8 MHz to 53.8 MHz. Where the transition of state is changed from “0,0” to “1,1”, and the optical contrast is 96%. All peaks of signals in Fig. 3(a,b) satisfy enhancement condition ∆_1_ + λ_±_ + *λ*_+±_ + *λ*_++±_ = 0. In contrast to left peaks, the shape of right peaks is smaller in Fig. 3(a) and is larger in Fig. 3(a), which is attributed to different cavity feedback dressing effect. In Fig. 3(c), Δ_*ac*_ is scanned at discrete Δ_2_ with all beams turned on and setting Δ_3_ = 0. When satisfying conditions Δ_1_ + Δ_2_ = 0, Δ_1_ − Δ_*ac*_ = 0 and Δ_1_ + Δ_3_ = 0, we can get the maximum value in Fig. 3(c1). From top to bottom, the increased detuning Δ_2_ result the decreased *n*_2_, and one can witness that the frequency offset Δυ in Fig. 3(c) is approximately 110 MHz. Where the change of ODB phenomena is very small due to saturation effect. Here the transition of state is similar to Fig. 3(b), but the optical contrast is 80%.

Same with Fig. 2, in Fig. 4, we discuss the output multi-mode states of the PTS and cavity mode FWM signal (***E***_*F*_) versus the frequency detuning of ***E***_3_ (Δ_3_). By changing frequency detuning of dressing fields, we realize the switch action between different output multi-mode states. Using the phenomenon of ODB, we investigate this action by analyzing the “∞”-shape non-overlapping region of folded signals. And same with Figs 2 and 3, the nonreciprocity of signals is also caused by different feedback dressing between two edges (rising and falling edges) in Fig. 4.

In Fig. 4(a,b), the curves represent ***E***_*F*_ signal by scanning the dressing detuning Δ3 with the external dressing effect of ***E***_3_. In Fig. 4(a), when Δ_1_ is decreased first in Fig. 4(a1–a3) then is increased in Fig. 4(a3–a5), the measured ***E***_*F*_ signal is obtained. Compared to Figs 2(a) and 3(e), the difference is that the signals in Fig. 4(a1,a2) have peaks and satisfy the enhancement condition ∆_1_ + λ_±_ + *λ*_+±_ = 0. While the condition of Fig. 4(a3–a5) change from semi-suppression and semi-enhancement to suppression (∆_1_ + ∆_3_ = 0). With the changing of Δ_1_, the frequency offset Δυ is bit-changed in Fig. 4(a1–a3) and then is decreased from 90.2 MHz to 35.6 MHz gradually. In this state, with L-state of PO, FD of Δ_1_ is changed from B-state to S-state and then to B-state again, and the state of FWM switches from “0,0” to “1,1” and then to “0,0” again. The optical contrast calculated in this case is 65%. With increased cavity detuning Δ_*ac*_ from top to bottom, the signals of ***E***_*F*_ as shown in Fig. 4(b). Where the peaks of signals satisfy the enhancement condition ∆_1_ + λ_±_ + *λ*_+±_ = 0, the change of degree of nonreciprocity ODB is similar with Fig. 3(b), but the state of FWM is keeping at “1,1” state with H-state of PO.

Figures 4(c,d) are signals of PTS corresponding to Fig. 4(a,b), respectively. In Fig. 4(c), the peaks of signals satisfy the enhancement condition ∆_1_ + λ_±_ + *λ*_+±_ = 0. From top to bottom, with changed Δ_1_, the enhancement of peaks is weakened first in Fig. 4(c1–c3) then is strengthened in Fig. 4(c3–c5), but the frequency offset Δυ is little-changed which is attributed to the enhancement effect. Here the transition of PTS state is contrary to Fig. 4(a). Compared with Fig. 4(c), the signals have dips in Fig. 4(d1,d2) that satisfy the suppression condition ∆_1_ + ∆_3_ = 0. Then, the condition of signals change from suppression to semi-suppression and semi-enhancement in Fig. 4(d2–d5). Besides, the degree of “∞”-shape non-overlapping region of ODB phenomena is decreased gradually due to changed suppression and enhancement conditions. Where the transition of state is changed from “0,0” to “1,1”, and the optical contrast is 96%.

In the Figs 2–4, the corresponding switching ratio of our flip-flop converter is attributed to the degree of non-overlapping region. If the size of non-overlapping region between dips or peaks in same baseline is big enough, it may be benefit for the division of two states. Such results can be exploited for frequency detuning division multiplexing. Here we use the channel equalization ratio $P=1-{({\sum }_{1}^{N-1}{({S}_{i}-S)}^{2}/S)}^{1/2}$ to measure the de-multiplexing effect, where ***s*** is the area of one dip or peak, ***s***_*i*_ is the area of non-overlapping region. In Fig. 3(a–c), *P* is near 100%, we shall obtain more balanced and stable spatial channels. In Figs 2(e,f), 3(d–f) and 4(a,c), the channel equalization *P* can approach 80–90%. We find that the high channel equalization ratio is caused by large of frequency offset Δυ.

## Conclusion

In summary, we study the realization of a dual-bistability flip-flop converter in cavity and PA-FWM. The flip-flop action results from the nonreciprocity ODB phenomena which is induced by self-dressing effect (cavity feedback dressing). The degree of this ODB can reflect by the “∞”-shape non-overlapping region of folded signals, which is changed with different input parameters. Therefore, with high optical contrast and stable spatial channels (channel equalization ratio), we can obtain many kinds of output multi-mode states by controlling the input parameters. By switching the states of PO or FD, we also can realize the convert of output multi-mode states. Specifically, the switch speed of this flip-flop converter is about 16 ns. These results are well explained with theoretical model. The observed phenomenon has novel and promising development for generation and potentially applicable in all-optical devices and quantum information processing.

## Methods

### Experimental setup

The experiment is performed in a composite atom-cavity system which contains a four-level ^85^Rb atomic vapor cell in the optical ring cavity as shown in Fig. 1(a). With the length of 38 cm, the cavity is consisted of a plate mirror M3 (with reflectivity of 97.5% at 780 nm) and two plate-concave mirrors (M1 99.9% and M2 97.5% at 780 nm). M1 is mounted on a PZT for adjusting and locking the cavity length. In the setup, a weak probe beam ***E***_1_ counter-propagates with a strong pumping beam ***E***_2_. Another pumping beam ***E***_2’_ propagates along the optical axis of the cavity (indicated by the dashed line) having an angle of 2° with ***E***_2_. Hence a phase conjugate FWM signal ***E***_*F*_ propagates in the opposite direction of ***E***_2’_, which means the signal of ***E***_*F*_ is mode-matched to the cavity and can form cavity mode. The external-dressing beam ***E***_3_ propagates at ***E***_2_ direction. All incident beams are focused at the center of the optical cavity by optical lenses. The temperature of the cell is set to 75 °C in order to have enough atoms in the cavity to enhance the strength of atom-cavity coupling. The cavity transmission spectrum of ***E***_*F*_ leaked from M3 (FWM) is detected by avalanche photodiode detector (APD), and the absorption of ***E***_1_ (PTS) is detected by the other APD.

### Evolution of the cavity field and the density matrix operators

Under the weak-cavity field limitation and with all the atoms initially in the ground state |0〉, the evolution of the cavity field and the density matrix operators obey the following linear equations as:

7 $$\dot{a}=-[i({{\rm{\Delta }}}_{1}-{{\rm{\Delta }}}_{ac})+\gamma ]a+ig\sqrt{N}{\rho }_{10}$$

8 $${\dot{\rho }}_{10}=-[i{{\rm{\Delta }}}_{1}+{{\rm{\Gamma }}}_{10}]{\rho }_{10}+i{G}_{F}{\rho }_{00}+ig\sqrt{N}a{\rho }_{00}+i{G}_{2}^{\ast }{\rho }_{20}+i{G}_{3}{\rho }_{30}$$

9 $${\dot{\rho }}_{20}=-[i({{\rm{\Delta }}}_{1}+{{\rm{\Delta }}}_{2})+{{\rm{\Gamma }}}_{20}]{\rho }_{20}+i{G}_{2}{\rho }_{10}$$

10 $${\dot{\rho }}_{30}=-[i({{\rm{\Delta }}}_{1}{+{\rm{\Delta }}}_{3})+{{\rm{\Gamma }}}_{30}]{\rho }_{30}+i{G}_{3}^{\ast }{\rho }_{10}$$

where the over-dots represent the first-order derivative with respect to time *t*; *γ* is the decay rate for the cavity; Δ_*ac*_ = *ω*_10_ − *ω*_*c*_ is the detuning of the cavity field with cavity resonant frequency *ω*_*c*_, ${G}_{F}\propto \sqrt{2/{\varepsilon }_{0}2c\hslash }N{\mu }^{2}{\rho }_{F2}^{(3)}$, $g\sqrt{N}$ results from the resonant florescence induced by ***E***_1_ and treated as the strength of atom-cavity coupling with single-atom-cavity coupling strength g and atom number N, and the atom-cavity coupling strength has a similar dressing effect with internal-dressing effects of ***E***_1_, ***E***_2_, ***E***_2’_ and external-dressing effect of ***E***_3_.

### The photon numbers of the output Stokes and anti-Stokes fields

With the amplification of OPA-FWM process, the photon numbers of the output Stokes and anti-Stokes fields with ***E***_1_ injection are described as:

11 $${N}_{S}=\langle {\hat{a}}_{out}^{+}{\hat{a}}_{out}\rangle =\frac{1}{2}[\,\cos (2t\sqrt{AB}\,\sin \,\frac{{\varphi }_{1}+{\varphi }_{2}}{2})+\,\cosh (2t\sqrt{AB}\,\cos \,\frac{{\varphi }_{1}+{\varphi }_{2}}{2})]{|\alpha |}^{2}.$$

12 $${N}_{aS}=\langle {\hat{b}}_{out}^{+}{\hat{b}}_{out}\rangle =\frac{1}{2}\frac{B}{A}[\,\cosh (2t\sqrt{AB}\,\cos \,\frac{{\varphi }_{1}+{\varphi }_{2}}{2})-\,\cos (2t\sqrt{AB}\,\sin \,\frac{{\varphi }_{1}+{\varphi }_{2}}{2})]{|\alpha |}^{2}.$$

where ${\hat{a}}^{+}(\hat{a})$ and ${\hat{b}}^{+}(\hat{b})$ attribute to the Stokes and anti-Stokes fields, respectively; ${|\alpha |}^{2}=\pi {\varepsilon }_{0}c\hslash {({G}_{{\rm{AF}}}r/{\mu }_{10})}^{2}/2{\omega }_{1}$ denotes the intensity of PA-FWM, *r* is the radius of the probe field, *G*_*AF*_ is the PA-FWM amplification factor, and ${G}_{{\rm{A}}F}\propto \sqrt{2/{\varepsilon }_{0}c\hslash }N{\mu }^{2}{\rho }^{(3)}$. The modulus A and B (phase angles *φ*_1_ and *φ*_2_) defined in ${\rho }_{01(S)}^{(3)}=A{e}^{i{\varphi }_{1}}$ and ${\rho }_{01(aS)}^{(3)}=B{e}^{i{\varphi }_{2}}$ for ***E***_*S*_ and ***E***_*aS*_, respectively.
